# A fatal outbreak of neonatal sepsis caused by *mcr-10*-carrying *Enterobacter kobei* in a tertiary care hospital in Nepal

**DOI:** 10.1016/j.jhin.2022.03.015

**Published:** 2022-04-20

**Authors:** S. Manandhar, Q. Nguyen, D.T. Pham, P. Amatya, M. Rabaa, S. Dongol, B. Basnyat, S.M. Dixit, S. Baker, A. Karkey

**Affiliations:** a Oxford University Clinical Research Unit, Patan Academy of Health Sciences, Kathmandu, Nepal; b Centre for Tropical Medicine and Global Health, Medical Sciences Division, Nuffield Department of Medicine, University of Oxford, Linacre College, Oxford, UK; c Oxford University Clinical Research Unit, Ho Chi Minh City, Vietnam; d Department of Pediatrics, Patan Academy of Health Sciences, Kathmandu, Nepal; e Center for Molecular Dynamics Nepal, Kathmandu, Nepal; f Cambridge Institute of Therapeutic Immunology & Infectious Disease, Department of Medicine, University of Cambridge, Cambridge, UK

**Keywords:** *Enterobacter kobei*, Sepsis outbreak, NICU, Whole-genome sequencing, Colistin-resistance, *mcr-10*

## Abstract

**Background:**

*Enterobacter kobei* is an emerging cause of outbreak of nosocomial infections in neonatal intensive care units (NICUs). Between July and September 2016, a NICU in a tertiary care hospital of Nepal observed an abrupt increase in the number of neonatal sepsis cases caused by *Enterobacter* spp. infecting 11 out of 23 admitted neonates, five of whom died of an exacerbated sepsis.

**Aim:**

To confirm the suspected outbreak, identify environmental source of infection, and characterize genetic determinants of antimicrobial resistance (AMR) and virulence of the pathogen.

**Methods:**

Whole-genome sequencing of all *Enterobacter* spp. isolated from blood cultures of septic neonates admitted to NICU between May 2016 and December 2017 was performed. Also, an environmental sampling was intensified from fortnightly to weekly during the outbreak.

**Findings:**

The genomic analysis revealed that 10 out of 11 non-duplicated *E. kobei* isolated from neonatal blood cultures between July and September 2016 were clonal, confirming the outbreak. The isolates carried AMR genes including *bla*_AmpC_ and *mcr-10* conferring reduced susceptibility to carbapenem and colistin respectively. The environmental sampling, however, failed to isolate any *Enterobacter* spp. Reinforcement of aseptic protocols in invasive procedures, hand hygiene, environmental decontamination, fumigation, and secluded care of culture-positive cases successfully terminated the outbreak.

**Conclusion:**

Our study underscored the need to implement stringent infection control measures to prevent infection outbreaks. For the first time, we report the emergence of carbapenem and colistin non-susceptible *E. kobei* carrying *mcr-10* gene as a cause of nosocomial neonatal sepsis in a NICU.

## Introduction

Neonates with critical health conditions often require specialized hospital care in neonatal intensive care units (NICUs). Vulnerable physiological condition of neonates together with the routine use of invasive medical procedures make the NICU admitted neonates prone to acquire infections, and make NICUs the hotspots for infection outbreaks [1−3]. There are several reports on infection outbreaks in NICUs globally [4−6].

*Enterobacter* spp. are important opportunistic pathogens. The ability of *Enterobacter* spp. to adapt in the hospital environment due to their intrinsic resistance to several clinically used antimicrobials along with the ability to readily acquire mobile genetic elements conferring virulence and antimicrobial resistance (AMR) make them increasingly important nosocomial pathogens associated with outbreak potentials [7]. Multidrug-resistant (MDR) strains of *Enterobacter* spp. are commonly associated with outbreaks among immune-compromised individuals such as NICU admitted neonates [3,4,6,8]. Such outbreaks are often associated with increased risk of treatment failure, and high morbidity and mortality thereby imposing a significant challenge to the hospital [1,3]. Under outbreak investigation using several biotyping and genotyping methods, whole-genome-based single nucleotide poly-morphism (SNP) analysis offers the highest resolution to confirm and characterize transmission dynamics of the pathogens.

Patan hospital had a history of several recurrent NICU out-breaks with a high case fatality rate [8−10]. In mid-2016, an *Enterobacter* spp.-driven fatal sepsis outbreak was suspected to have occurred in the NICU, where 48% (11/23) of the admitted neonates were infected in a three-month period, five of whom died of an exacerbated sepsis. In this study, whole-genome sequencing (WGS) was performed on clinical isolates of *Enterobacter* spp. isolated during the suspected period, and an outbreak due to *E. kobei* with reduced susceptibility to imipenem and colistin was identified retrospectively.

## Methods

### Study setting

This study was carried out in Patan hospital, one of the largest tertiary care hospitals located in Lalitpur district of Kathmandu, Nepal. The hospital has a level-3 NICU unit with eight beds to provide an ICU care to the inborn neonates. An adjacent paediatric intensive care unit (PICU) provides an ICU care to the outborn neonates and children. The neonates requiring a non-intensive medical care are admitted to the clean nursery if they are devoid of any clinical signs of, or risk factors for, sepsis; else are admitted to the septic nursery. The nurseries lie next to the NICU.

### Ethical approval

This study was a part of a prospective observational cohort study conducted from May 2016 until December 2017 in a level-3 NICU of Patan hospital to understand the risk factors of acquiring neonatal sepsis in a NICU. Ethical approval was obtained from Nepal Health Research Council (NHRC) under an approval registration number of 278/2015, and from Oxford Tropical Research Ethics Committee (OxTREC) under registration number of 24e16 for the research titled ‘Prevalence of ESBL producing Enterobacteriaceae and the probable risk factors of hospital acquired neonatal infections in the NICU of Patan hospital’.

All NICU admitted inborn neonates for whom their guardians provided a written informed consent were enrolled in the study. The neonates were followed up daily during their stay in NICU until discharge or death. All necessary diagnostic and therapeutic care was administered to the neonates as a part of routine clinical care as per the NICU protocol. Relevant data on clinical, therapeutic, and laboratory investigations were recorded in a pre-tested case report form by referring to the clinical notes marked by the paediatrician.

### Study definition and preface of the situation

An outbreak of an infection was defined when two or more NICU-admitted neonates yielded the same bacterial species sharing identical or similar antimicrobial susceptibility test (AST) profile in one or more blood cultures per unit time.

The number of septic neonates that were culture positive for *Enterobacter* spp. increased sharply to 50% (6/12) of monthly admissions in August 2016 from the preceding 17% (1/6) in July 2016 and 0% in each June (0/10) and May (0/12) 2016. This raised strong suspicion of an onset of *Enterobacter* spp.-driven infection outbreak in NICU. To determine the genetic relatedness of bacterial pathogens and to understand the epidemiology of the suspected outbreak, WGS of all *Enterobacter* spp. isolated from blood culture of septic neonates in NICU was performed between May 2016 and December 2017. To obtain a better overview of the outbreak, further blood isolation of *Enterobacter* spp. after neonates had been discharged to other wards was also included in WGS.

### Microbiological investigation

Upon clinical suspicion of sepsis, 1e2 mL of peripheral blood was drawn from the neonate, aseptically inoculated into BD-Bactec Peds plus/F culture vials (Becton Dickinson, Plymouth, UK) and incubated in an automated BD Bactec FX40 culture system. When indicated as culture positive, a small volume of blood sample was aseptically aspirated from the culture vial to be inoculated on to 5% sheep blood agar, MacConkey agar, and chocolate agar. The culture plates were incubated at 35 ± 2 °C for 18–24 h. Bacterial identification was carried out manually using the standard microbiological methods [11]. The Gram-negative bacilli that were lactose-fermenting, motile, indole-negative, urease-negative, citrate-positive, hydrogen sulphide non-producing, and giving acid/acid reaction with gas production in triple sugar iron test were identified phenotypically as *Enterobacter* spp. The isolates were subjected to antimicrobial susceptibility testing (AST) by modified KirbyeBauer disc diffusion method [12]. The AST for colistin was performed by determining minimum inhibitory concentration (MIC) using the E-test method. The AST results were interpreted following the breakpoint guidelines of the Clinical and Laboratory Standards Institute [13].

### Environmental screening

During the outbreak, the routine fortnightly conducted environmental screening was intensified to weekly from mid-August 2016 until October 2016. Various environmental samples (Supplementary Table S1) were collected from high-touch surfaces of NICU as recommended by the hospital infection prevention and control (IPC) committee. A sterile individually packed cotton swab was taken, immersed in sterile normal saline, and horizontally wiped across a given sampling surface for 10–30 s. The moist swab was returned to the container and transported to the microbiology laboratory at room temperature. The swab was inoculated on to MacConkey agar (Mast Group Ltd, Bootle, UK) and 5% blood agar (Mast Group Ltd), and incubated at 35 ± 2 °C for 18–24 h. The bacterial identification was carried out manually using the standard microbiological methods [11].

### Infection control measures

During the outbreak period, the existing NICU infection control protocol was reviewed and reinforced with consultation from the hospital IPC committee. Specific hand hygiene measures were reinforced to all NICU staff and parents/guardians of neonates. All staff were required to watch the hand washing video every three months and were audited regularly. Aseptic preparation of intravenous (i.v.) fluid/medication using sterile gloves, disinfecting the vial surface before and after drawing, avoiding use of same i.v. bottle for more than 24 h, using separate bottles for each patient, and avoiding pooling antibiotics were implemented. Aseptic invasive procedure was practised, including use of maximum aseptic barrier, disposable ventilator circuit, and skin preparation with alcohol, iodine, followed by chlorhexidine. All devices and equipment were appropriately disinfected or sterilized before and after the use. Essential daily care items, such as measuring tape, stethoscope, fixation tape, artery forceps, scissors, thermometer, etc., were allocated individually for each neonate by their cot side. The culture-positive neonates were cohort nursed. Daily cleaning with Klorkleen solution (Medentech, Wexford, Ireland) in 200 ppm final chlorine was used once every nursing shift, whereas that of 1000 ppm was used fortnightly; fumigation was performed after suspected outbreak.

### Whole-genome sequencing

The genomic DNA of all 18 *Enterobacter* spp. isolated from May 2016 to December 2017 was extracted using the Wizard Genomic DNA Extraction Kit (Promega, Madison, Wisconsin, USA). The dual index-tagged pooled DNA libraries were pre-pared using Nextera XT DNA Library Preparation Kit (Illumina, San Diego, CA, USA). WGS was performed on a HiSeq X Ten platform (Illumina) to generate 150 bp paired-end reads. Raw Illumina reads were deposited in European Nucleotide Archive (ENA) under the accession numbers as described in Supplementary Table S2.

### Genomic analysis

After cleaning the raw Illumina reads, SRST2 v0.2.0 was used to identify AMR genes, virulence factors, plasmid replicons, and multi-locus sequence type (MLST) using the following respective databases: ARG-ANNOT, BIGSdb, PlasmidFinder, and *Enterobacter cloacae* complex MLST typing scheme (https://pubmlst.org/mlst/organisms/enterobacter-cloacae) [14−17]. The cleaned reads were assembled *de novo* using Unicycler v0.48 followed by rapid annotation using Prokka v1.5 [18,19].

### Species identification

The taxonomic assignment of *Enterobacter* spp. is complex. Recently, a genomic-based average nucleotide identity (ANI) tool has been used to provide an updated taxonomy of *Enterobacter* genus. In this study, a fast approximate ANI tool, called MASH v1.1.1, was used to generate a pairwise ANI-based distance matrix between our 18 *Enterobacter* spp. isolates and the published reference collection of 22 *Enterobacter* spp. [20,21]. A pairwise ANI with a cut-off threshold of 96% was used to identify appropriate species.

### Phylogenetic analysis

To reconstruct the phylogenetic relation of 11 non-duplicated *E. kobei* outbreak isolates, the reads were mapped against the reference genome, *E. kobei* strain C16 (accession number: CP042578), using RedDog pipeline v1.10b (https://github.com/katholt/RedDog) and SNPs were identified with SAMTools v1.3.1. High-quality SNPs were extracted using a standard approach [22,23]. Using Gubbins v1.4.5, the SNPs identified in recombinant regions were removed, and a final alignment of 13 SNPs was created to construct a maximum likelihood (ML) phylogenetic tree using IQ-TREE v1.4.4 with the best-fit evolutionary model (F81+ASC) identified based on Bayesian information criterion in jModelTest [22,24]. Support for the ML tree was assessed via 1000 pseudo-replicates.

## Results

### *Species identification of* Enterobacter *spp*.

Overall, 18 *Enterobacter* spp. were isolated during the study period of May 2016 to December 2017. Of these, the ANI-based taxonomic analysis showed that all 14 phenotypically identified *Enterobacter* spp. obtained during the suspected outbreak period (July to September 2016) belonged to *E. kobei* spp., whereas the remaining four *Enterobacter* spp. isolated in 2017 after the outbreak belonged to non-*kobei* species (Table I).

### Temporal description of the outbreak

**Figure 1:**
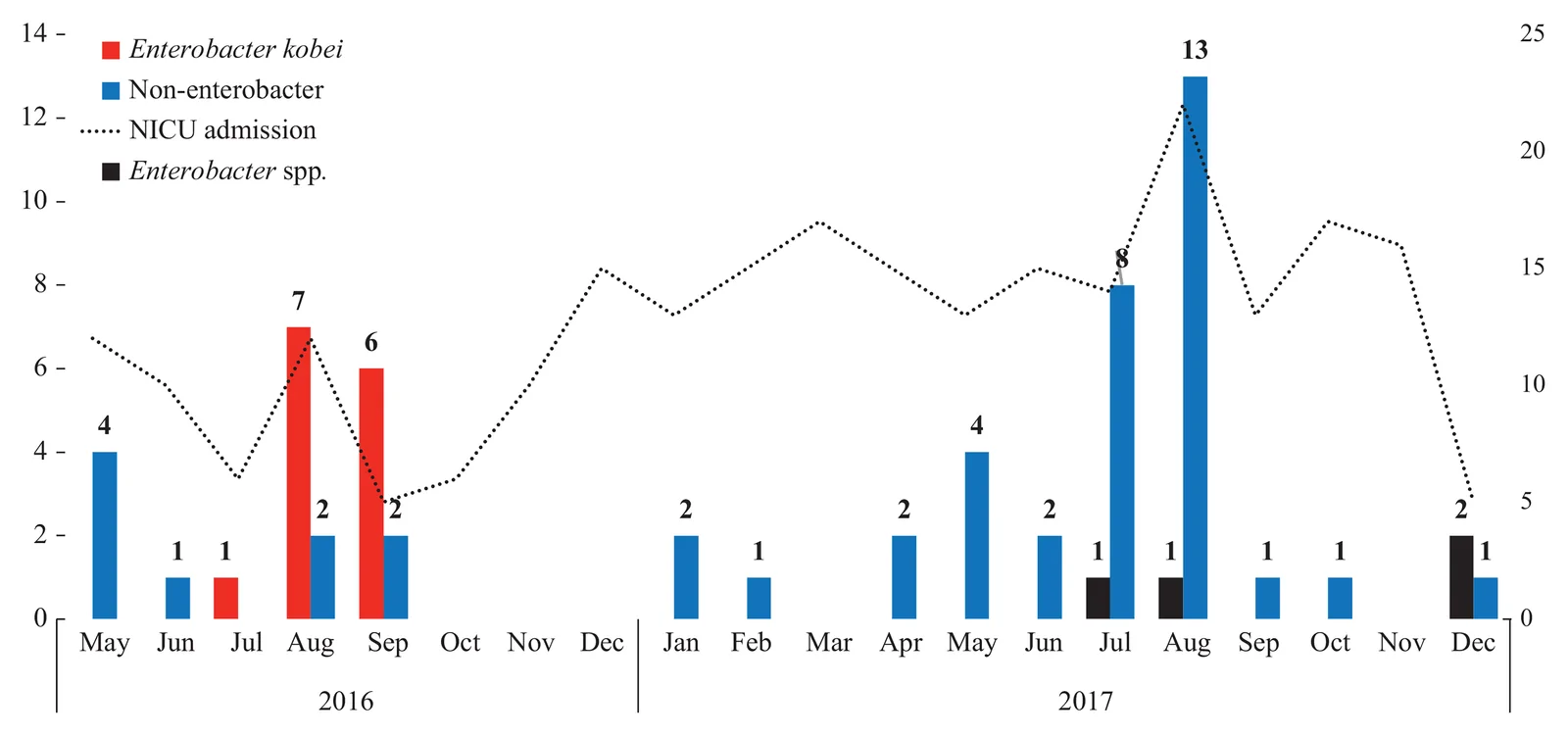
Numbers of *E. kobei* (red bars), *Enterobacter* spp. (black bars), and non-*Enterobacter* spp. (blue bars) isolated from blood samples of the neonates admitted to the neonatal wards (dotted line) between May 2016 and December 2017.

**Figure 2:**
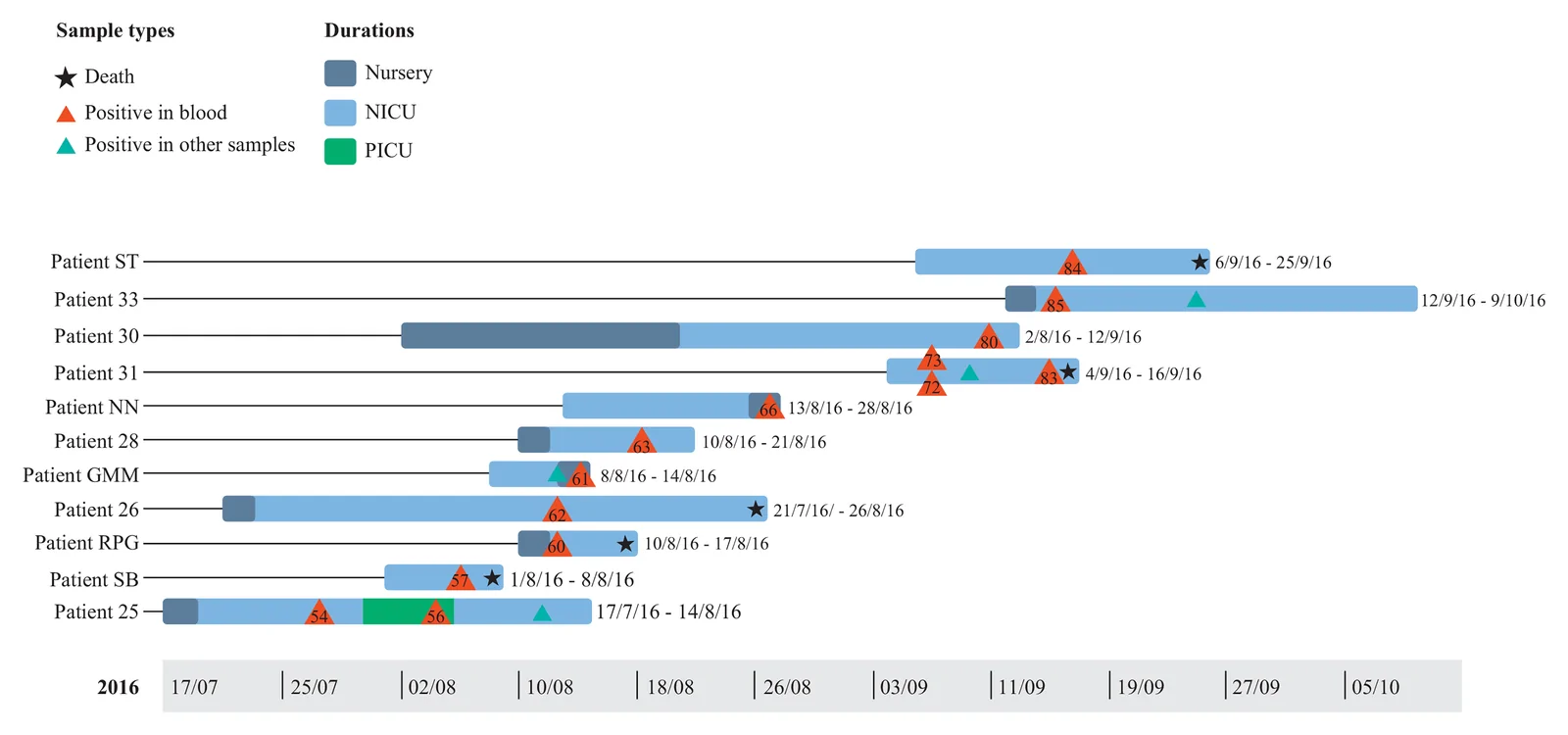
Chronological development of E. kobei-driven neonatal sepsis outbreak at Patan Hospital NICU between July and October 2016, shown by individual neonates. NICU, neonatal intensive care unit; PICU, paediatric intensive care unit.

In May and June 2016, there was no isolation of any *Enterobacter* spp. from NICU. In July 2016, *E. kobei* was detected for the first time from one of six NICU admitted neonates. Notably, in the following month (August 2016), *E. kobei* isolation spiked when half of the NICU admitted neonates (6/12) were subsequently infected by it, raising strong suspicion of an outbreak. In the next month also (September 2016), new infections due to *E. kobei* continued when 80% of admitted neonates (4/5) were infected (Figure 1). Overall, during these three months (July to September 2016), 11 out of 23 (48%) NICU admitted neonates were infected by *E. kobei*, of which five neonates died due to sepsis. No *Enterobacter* spp. isolates were detected during the subsequent nine months. In July, August, and December of 2017, four non-*kobei* isolates of *Enterobacter* spp. (*E. cloacae, E. xiangfangensis*, and *E. mori*) were cultured from blood samples of the septic neonates; however, *E. kobei* was no longer isolated from the NICU-admitted neonates. Figure 2 illustrates a case-by-case chronological development of *E. kobei*-driven sepsis outbreak. The epidemiological and clinical features of neonates infected with *E. kobei* during the outbreak are shown in Supplementary Table S3.

### *Phylogenetics and gene content analysis of* E. kobei *outbreak strains*

The phylogenetic analysis of 11 non-duplicate *E. kobei* isolated between July and September of 2016 gave a strong indication for an outbreak of neonatal sepsis in NICU (Supplementary Figure S1A). All outbreak isolates formed a tight cluster separated by 13 core SNPs. Of these, isolate 56, which was recovered from the patient 25 during a temporary transfer to PICU, was the most distant from the remaining isolates with a phylogenetic distance of 5 SNPs. Among the remaining 10 isolates, the average pairwise SNP distance was 3 (ranging from 0 to 5).

Ten out of 11 outbreak isolates had an identical but limited set of AMR genes including *bla*_AmpC_, *fosA2, bla*_*ACT-28*_, and *mcr-10*, predicted to confer resistance to β-lactams, fosfomycin, carbapenem, and colistin respectively. The phenotypic susceptibility test of 10 outbreak isolates demonstrated reduced Figure 2. Chronological development of *E. kobei*-driven neonatal sepsis outbreak at Patan Hospital NICU between July and October 2016, shown by individual neonates. NICU, neonatal intensive care unit; PICU, paediatric intensive care unit. susceptibility (intermediate or, resistant) to amoxicillin (10/10, 100%), cefotaxime (9/10, 90%), imipenem (8/10, 80%), and colistin (80%, 8/10) (colistin MICs ranging from 2 to 4 mg/L; Table I). The isolates carried *mrkABCF* and *fliA* virulence genes, encoding for formation of biofilm, and flagellum-specific sigma factor respectively. By contrast, *E. kobei* isolate 56 carried an extensive array of AMR genes conferring resistance to third-generation cephalosporins (*bla*_ACT-28_, *bla*_CTXM-1_, *bla*_TEM-1D_), aminoglycosides (*aac3-IIa, aacA-ad, atrA/B*), phenicols (*catA1/B4*), quinolones (*qnrB1*), trimethoprim (*dfrA5)*, fosfomycin (*fosA2*), tetracycline (*tetAB*), and colistin (*mcr-10*) (Supplementary Figure S1A). Isolate 56 also carried several virulence genes, including *hcp/tssD* and *iroN* encoding for T6SS toxin secretory system and salmochelin siderophore, respectively.

### Results of environmental screening

Supplementary Table S1 summarizes the microbiological results of various environmental samples collected during the outbreak to identify any potential environmental source of infection. Though *Acinetobacter* spp. and non-pathogenic bacteria such as *Bacillus* and *Micrococcus* spp. were occasionally isolated, *Enterobacter* spp. was not isolated from any environmental samples during the outbreak period.

## Discussion

NICU remains a hotspot of infection outbreaks [2,3]. Our NICU had a history of multiple outbreaks primarily caused by *Klebsiella pneumoniae* and *Enterobacter* spp. [8−10]. As never evidenced before, the emergence of *E. kobei* as a cause of neonatal sepsis in our NICU was intriguing. *E. kobei* has been seldom reported in clinical specimens, such as bronchoalveolar lavage, blood, and abscess [25,26]. However, to date, there have been no published reports on *E. kobei* as a cause of neonatal sepsis. This may be because of two main reasons. First, the precise species differentiation of *Enterobacter cloacae* complex bacteria using conventional method is challenging due to the complex and evolving taxonomic assignment of *Enterobacter* spp., leading to limited clinical reports on *E. kobei* [7]. Second, *E. kobei* may have recently evolved to become an emerging cause of nosocomial infections in immunocompromised individuals, such as neonates, in high-dependency hospital units.

Detection of *E. kobei* as a cause of sepsis outbreak suggested a potential establishment and persistence of this pathogen in our NICU environment, although direct person-to-person transmission cannot be excluded. The fact that all outbreak isolates had the genes encoding for type 3 fimbriae and flagella, facilitating formation of biofilm, suggests that *E. kobei* may be able to persist in hospital surfaces via biofilm formation [27−29]. This may explain the history of recurrent infection outbreaks in our unit with an apparent cessation only after an extensive environmental fumigation. The possibility of environmental colonization as a source of infection is further corroborated by the fact that, during the outbreak, not all neonates were infected simultaneously but were infected intermittently during a span of three months, and the genetic diversity among the outbreak strains was limited. Our findings indicate that the majority of infections might have been acquired horizontally directly, or indirectly from one or more common environmental or biological sources. A longitudinal study conducted during the same time-period in this hospital’s NICU indicated compromise in hospital IPC measures contributing to the horizontal transmission of infection via nosocomial exposures, such as increased use of invasive procedures such as mechanical ventilation, umbilical artery catheter, umbilical vein catheter, and intravenous catheter [30]. A persistent source of infection existing in the environment coupled with compromise in the hospital IPC measures most likely drove the observed infection outbreak in the unit during the given period. Further experimental studies are required to measure the ability of *E. kobei* in formation of biofilms.

With increasing evidence that contaminated hospital sur-faces can contribute to nosocomial infections and outbreaks, the role of environmental surveillance is indispensable in investigating the source of infectious outbreaks [8]. In this study, the environmental surveillance of frequently touched NICU surfaces, however, could not pinpoint the source of *E. kobei* colonization. This may be because the source of bacterial colonization was too elusive to be captured in our sampling protocol, or because our sampling method was insufficiently sensitive to the target pathogen. However, other bacterial isolates, such as *Acinetobacter* spp., *Bacillus, Micrococcus*, and coagulase-negative staphylococci were successfully cultured using the same method. The use of other sampling tools such as macro-foam swab, sponge, or polywipe, along with the use of transport media, and pre-enrichment before direct inoculation might have improved the detection of the target pathogen. An earlier study from our unit reported performing a rigorous sampling and surveillance culture of NICU healthcare workers during an NDM-1-positive *K. pneumoniae*-linked sepsis outbreak in late 2011 [9]. Though *K. pneumoniae* was isolated from several samples, none was ESBL-producing, thus yielding inconclusive results. Another study conducted in the same unit in late 2012 genomically identified the soap container by a NICU sink to be one of the sources of an *E. cloacae* outbreak, though the source for other clusters could not be identified [8].

During our study period, the IPC measures were revised and rigorous multi-disciplinary infection control interventions were implemented from mid-August 2016. Our measures included an extensive environmental decontamination; reinforcement of hand hygiene practice followed by periodic audit; a standard aseptic protocol for invasive procedures; allocation of daily care essentials for each neonate by their cot side; use of disposable ventilator circuit; proper decontamination of equipment before being shared to others; and an allocation of dedicated nursing staff for clinical care of culture-positive cases. Adherence to stringent IPC measures helped to terminate further transmission events and potentially eliminated the source of infection, as no *E. kobei* was further detected during the study period from September 2016 to December 2017.

In our hospital, PICU provided an intensive care service primarily to the outborn neonates. Compared to those born and admitted within our hospital, infections among outborn neonates are expected to be caused by diverse and distinct bacterial aetiologies, possibly acquired from the community or prior healthcare institutions. Interestingly, we found that isolate 56 recovered from PICU was more distant from the remaining 10 outbreak isolates, and carried distinct AMR and virulence genes. We hypothesized that it might have been acquired from a different source in PICU, or it might have lost AMR and virulence gene cassettes during subsequent transmission events. Further, we speculate that different therapeutic management of outborn and inborn neonates may have imposed distinct selective pressure on the infecting organisms. This finding underscored the need to provide separate care for inborn and outborn neonates, and to monitor the potential spread of pathogens between these departments.

In conclusion, this study provided genomic evidence of a fatal outbreak of neonatal sepsis due to carbapenem and colistin non-susceptible *mcr-10*-carrying *E. kobei* in our NICU. Our study highlighted the importance of rigorous IPC measures and hospital surveillance to timely identify, contain, and prevent outbreaks. It also emphasized the need to segregate outborn from inborn neonates to prevent potential complexities of therapeutic management arising from distinct bacterial aetiologies, and to limit potential spread of bacterial pathogens between the two departments.

## Supplementary Material

**Appendix A. Supplementary data**

Supplementary data to this article can be found online at https://doi.org/10.1016/j.jhin.2022.03.015.

Supplementary Figure S1A, B

Supplementary Table S1, S2, S3

## Figures and Tables

**Table I:** Species, sequence type (ST), and AST results of *Enterobacter* spp. isolated from 14 neonates admitted to different neonatal units at Patan Hospital

Case ID	Isolate ID	Department	Species	ST	Phenotypic AST									
					AMX	CTX	CIP	COT	GEN	AMK	OFX	CHL	IMI	CS^a^
N_25_	54	NICU	*E. kobei*	NF	R	I	S	S	S	S	S	S	S	3
	56^b^	PICU	*E. kobei*	NF	R	R	I	S	R	I	S	R	S	3
N_SB_	57^b^	NICU	*E. kobei*	NF	R	R	I	S	R	I	S	R	S	3
N_RPG_	60^b^	NICU	*E. kobei*	NF	R	R	S	S	S	S	S	I	I	3
N_GMM_	61^b^	Nursery	*E. kobei*	NF	R	R	S	I	S	I	S	S	I	4
N_26_	62^b^	NICU	*E. kobei*	NF	R	I	S	S	S	S	S	R	I	3
N_28_	63^b^	NICU	*E. kobei*	NF	R	I	S	S	S	S	S	I	I	3
N_NN_	66^b^	Nursery	*E. kobei*	NF	R	R	S	S	S	S	S	I	I	3
N_31_	72	NICU	*E. kobei*	NF	R	S	S	S	S	S	S	S	I	2
	73^b^	NICU	*E. kobei*	NF	R	S	S	S	S	S	S	S	R	2
	83	NICU	*E. kobei*	NF	R	R	I	S	S	S	S	I	I	2
N_30_	80^b^	NICU	*E. kobei*	NF	R	R	S	S	S	S	S	R	S	2
N_33_	85^b^	NICU	*E. kobei*	NF	R	R	S	S	S	S	S	I	I	4
N_ST_	84^b^	NICU	*E. kobei*	NF	R	R	S	S	S	S	S	R	I	3
N_l23_	152	NICU	*E. cloacae*	167	R	S	S	S	S	S	S	S	S	2
N_l34_	153	NICU	*E. cloacae*	167	R	R	R	R	R	S	S	R	S	2
N_RK_	154	NICU	*E. xiangfangensis*	NF^#^	R	R	R	R	R	S	S	S	S	3
	155	NICU	*E. mori*	NF^##^	S	S	S	S	S	S	S	S	S	2

AMK, amikacin; AMX, amoxicillin; AST, antimicrobial susceptibility test; CHL, chloramphenicol; CIP, ciprofloxacin; COT, cotrimoxazole; CS, colistin; CTX, cefotaxime; GEN, gentamycin; I, intermediate; ID, identification number; IMI, imipenem; NF, not found; NICU, neonatal intensive care unit; OFX, ofloxacin; PICU, paediatric intensive care unit; R, resistant; S, susceptible; ST, sequence type. NF, NF^#^, and NF^##^ had distinct and unique allelic profiles not found in the reference database.

a For colistin, MIC (mg/L) values obtained by E-test are shown.

b Indicates 11 non-duplicate *E. kobei* isolates subsequently included in the phylogenetic analysis.
