# Supplementary material for: A fatal outbreak of neonatal sepsis caused by mcr-10-carrying Enterobacter kobei in a tertiary care hospital in Nepal
Source: J Hosp Infect. Author manuscript; Available in PMC 2026 Oct 16. (PMC7619562; doi:10.1016/j.jhin.2022.03.015)
Supplement: Supplementary file 1 [file EMS219683-supplement-Supplementary_Figure_S1A__B.pdf]

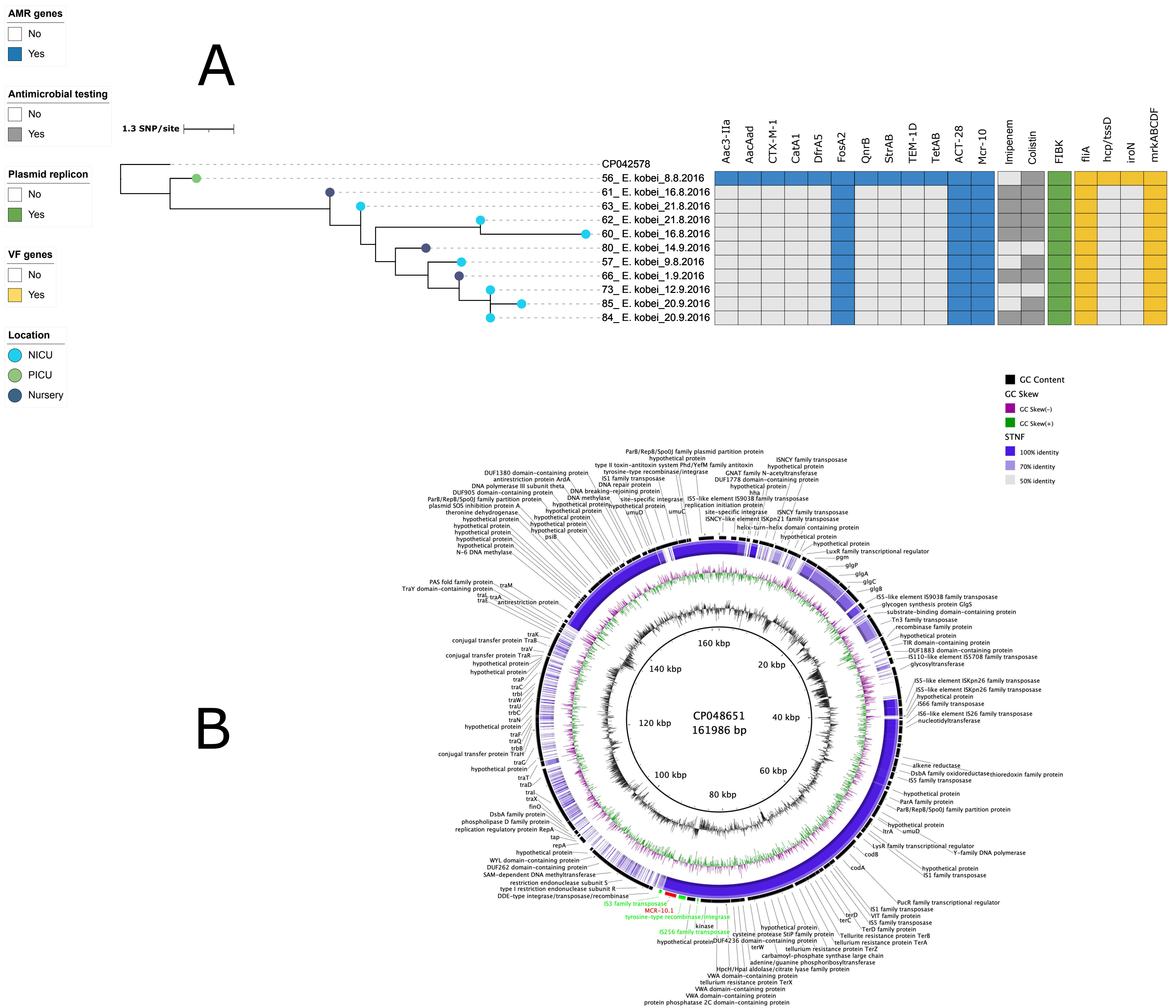

A
AMR genes
No
Yes
Antimicrobial testing
No
Yes
Plasmid replicon
No
Yes
VF genes
No
Yes
Location
NICU
PICU
Nursery
1.3 SNP/site
CP042578
56_ E. kobei_8.8.2016
61_ E. kobei_16.8.2016
63_ E. kobei_21.8.2016
62_ E. kobei_21.8.2016
60_ E. kobei_16.8.2016
80_ E. kobei_14.9.2016
57_ E. kobei_9.8.2016
66_ E. kobei_1.9.2016
73_ E. kobei_12.9.2016
85_ E. kobei_20.9.2016
84_ E. kobei_20.9.2016
Aac3-IIa
AacAad
CTX-M-1
CatA1
DfrA5
FosA2
QnrB
StrAB
TEM-1D
TetAB
ACT-28
Mcr-10
Imipenem
Colistin
FIBK
fliA
hcp/tssD
iroN
mrkABCDF
B
GC Content
GC Skew
GC Skew(-)
GC Skew(+)
STNF
100% identity
70% identity
50% identity
CP048651
161986 bp
160 kbp
20 kbp
40 kbp
60 kbp
80 kbp
100 kbp
120 kbp
140 kbp
MCR-10.1
IS3 family transposase
tyrosine-type recombinase/integrase
IS256 family transposase
