# Supplementary material for: A fatal outbreak of neonatal sepsis caused by mcr-10-carrying Enterobacter kobei in a tertiary care hospital in Nepal
Source: J Hosp Infect. Author manuscript; Available in PMC 2026 Oct 16. (PMC7619562; doi:10.1016/j.jhin.2022.03.015)
Supplement: Supplementary file 2 [file EMS219683-supplement-Supplementary_Table_S1__S2__S3.docx]

**Supplementary Figure S1.** The phylogenetic tree (A) and an *mcr-10*-carrying plasmid structure (B) of *E. kobei* causing neonatal sepsis at the neonatal intensive care unit and other neonatal departments at Patan Hospital. (A) Phylogeny tree rooted with reference *E. kobei* strain C16 used as an outgroup. The node colours correspond to different neonatal departments of bacterial isolation. The tip labels correspond to *E. kobei* isolates indicated the by ID numbers, also shown with the dates of isolation. The scale bar estimates the number of single nucleotide polymorphisms per site. The heat map shows the presence (blue, dark grey, green, and yellow colour) or absence (light grey colour) of the antimicrobial resistance genes, phenotypic antimicrobial resistance, plasmid replicon type, and virulence genes. (B) BLASTN comparison between IncFIBK plasmid identified from *E. kobei* isolate, and a reference plasmid from *E. roggenkampii* (accession number CP048651). The outside ring indicates the gene annotation of reference sequence. *Mcr-10* gene has been highlighted in red, and its mobile insertion sequence elements are coloured in green.

**Supplementary Table S1**

Summary of microbiological results of various NICU environmental samples collected weekly from August to October 2016.

| Swab samples | Microbiological results* |
| --- | --- |
| Drinking water tap | No growth after 48 hours of incubation |
| Ventilator | No growth after 48 hours of incubation |
| Feeding trolley | No growth after 48 hours of incubation |
| Big trolley | No growth after 48 hours of incubation |
| Door handle | No pathogen isolated after 48 hours of incubation |
| Infusion pump | No growth after 48 hours of incubation |
| Suction jar | No growth after 48 hours of incubation |
| Monitor | No growth after 48 hours of incubation |
| Floor-1 | No pathogen isolated after 48 hours of incubation |
| Floor-2 | No pathogen isolated after 48 hours of incubation |
| Floor-3 | Light growth of *Acinetobacter* spp. |
| Syringe pump | No growth after 48 hours of incubation |
| Warmer | No growth after 48 hours of incubation |
| Ambu bag | No growth after 48 hours of incubation |
| Nursing station desk-1 | Light growth of *Acinetobacter* spp. |
| Nursing station desk-2 | No pathogen isolated after 48 hours of incubation |
| Weigh machine | No pathogen isolated after 48 hours of incubation |
| Laryngoscope blade | No growth after 48 hours of incubation |
| Overhead warmer | No growth after 48 hours of incubation |
| Ventilator baby log | No growth after 48 hours of incubation |
| Phillips ventilator | No growth after 48 hours of incubation |
| Formula milk feed | No growth after 48 hours of incubation |
| Green hand washing tap | Heavy growth of *Acinetobacter* spp. |
| Phototherapy | No growth after 48 hours of incubation |
| Medicine trolley | No pathogen isolated after 48 hours of incubation |
| Reusable SpO2 probe | No pathogen isolated after 48 hours of incubation |
| Nasal swab-1 | Heavy growth of coagulase negative staphylococci |
| Nail bed (nail tips) | No growth after 48 hours of incubation |
| Nasal swab-2 | No pathogen isolated after 48 hours of incubation |
| Personnel hand swabs | No growth after 48 hours of incubation |
| NICU bed rail-3 | No growth after 48 hours of incubation |
| Bucket/Jug | Heavy growth of *Acinetobacter* spp. |
| Fan | No growth after 48 hours of incubation |
| Outer door handle | No pathogen isolated after 48 hours of incubation |
| Air conditioner | No pathogen isolated after 48 hours of incubation |

‘No pathogen isolated after 48 hours of incubation’ referred to the isolation of clinically insignificant bacteria, such as *Micrococcus* spp., *Bacillus* spp., coagulase negative staphylococci or poly-microbial growth after 48 hours of incubation.

**Supplementary Table S2**

List of raw Illumina reads deposited in European Nucleotide Archive (ENA) given with respective accession numbers.

| Isolate ID | Accession number | Study Code | Sample ID | Experiment number |
| --- | --- | --- | --- | --- |
| 85 | ERR6684058 | ERP131723 | ERS7302742 | ERX6309006 |
| 84 | ERR6684057 | ERP131723 | ERS7302741 | ERX6309005 |
| 80 | ERR6684056 | ERP131723 | ERS7302739 | ERX6309004 |
| 73 | ERR6684055 | ERP131723 | ERS7302738 | ERX6309003 |
| 66 | ERR6684054 | ERP131723 | ERS7302736 | ERX6309002 |
| 63 | ERR6684053 | ERP131723 | ERS7302735 | ERX6309001 |
| 62 | ERR6684052 | ERP131723 | ERS7302734 | ERX6309000 |
| 61 | ERR6684051 | ERP131723 | ERS7302733 | ERX6308999 |
| 60 | ERR6684050 | ERP131723 | ERS7302732 | ERX6308998 |
| 57 | ERR6684049 | ERP131723 | ERS7302731 | ERX6308997 |
| 56 | ERR6684048 | ERP131723 | ERS7302730 | ERX6308996 |

**Supplementary Table S3**

Summary on epidemiological and clinical features of neonates infected with *E. kobei* during the outbreak.

| Case ID | Gest age group | Birth weight group | NICU admission indication | Age on NICU admission (DOL) | Age on *E. kobei* isolation  (DOL) | NICU stay days | Final outcome |
| --- | --- | --- | --- | --- | --- | --- | --- |
| N_25_ | PT | LBW | PT/IUGR; symptomatic polycythaemia for possible exchange transfusion | 2 | 8  18 | 26 | Survived |
| N_SB_ | PT | VLBW | Severe respiratory distress | 1 | 6 | 7 | Dead |
| N_RPG_ | T | NBW | Persistent respiratory distress, congenital pneumonia | 1 | 3 | 7 | Dead |
| N_26_ | PT | LBW | Severe respiratory distress (tachypnoea> 90 breaths/min, nasal flaring, grunting), respiratory acidosis | 1 | 22 | 33 | Dead |
| N_GMM_ | T | NBW | Birth asphyxia | 1 | 6 | 4 | Survived |
| N_28_ | PT | LBW | Persistent respiratory distress | 1 | 8 | 12 | Survived |
| N_NN_ | PT | LBW | Severe respiratory distress | 1 | 15 | 12 | Survived |
| N_31_ | PT | LBW | Grunting with subcostal recession | 1 | 3  11 | 14 | Dead |
| N_30_ | PT | VLBW | Respiratory distress (occasional apnea, grunting, subcostal recession), congenital pneumonia | 20 | 40 | 23 | Survived |
| N_33_ | PT | LBW | Severe respiratory distress | 1 | 3 | 26 | Survived |
| N_ST_ | PT | VLBW | Severe respiratory distress with respiratory acidosis | 1 | 9 | 19 | Dead |

DOL, days of life; Gest, gestational; IUGR, Intra-uterine growth retardation; PPROM, prolonged premature rupture of membrane; PT, pre-term; T, term; VLBW, very low birth weight.
